# *Drosophila Nhe2* overexpression induces autophagic cell death

**DOI:** 10.1091/mbc.E24-02-0058

**Published:** 2024-07-01

**Authors:** Jobelle Peralta, Blake DuPriest, Daniel Orozco, Juan Reyna Pacheco, Laura Martins, Rachel Ann Soriano, Alan Wong, Ramy Wong, Bree Grillo-Hill

**Affiliations:** a Department of Biological Sciences, San José State University, San José, CA 95112; Hospital for Sick Children

## Abstract

Autophagy is a conserved catabolic process where double membrane-bound structures form around macromolecules or organelles targeted for degradation. Autophagosomes fuse with lysosomes to facilitate degradation and macromolecule recycling for homeostasis or growth in a cell autonomous manner. In cancer cells, autophagy is often up-regulated and helps cancer cells survive nutrient deprivation and stressful growth conditions. Here, we propose that the increased intracellular pH (pHi) common to cancer cells is sufficient to induce autophagic cell death. We previously developed tools to increase pHi in the *Drosophila* eye via overexpression of *DNhe2,* resulting in aberrant patterning and reduced tissue size. We examined fly eyes at earlier stages of development and found fewer interommatidial cells. We next tested whether this decrease in cell number was due to increased cell death. We found that the *DNhe2*-induced cell death was caspase independent, which is inconsistent with apoptosis. However, this cell death required autophagy genes, which supports autophagy as the mode of cell death. We also found that expression of molecular markers supports increased autophagy. Together, our findings suggest new roles for ion transport proteins in regulating conserved, critical developmental processes and provide evidence for new paradigms in growth control.

## Abstract (executive summary)

Cancer cells have constitutively increased intracellular pH (pHi), which is thought to facilitate cancer cell behaviors like increased proliferation and resistance to apoptotic cell death.

The authors show that overexpression of *Drosophila Nhe2* is sufficient to decrease cell number through autophagic cell death.

This suggests that increased pHi may induce autophagic cell death.

## INTRODUCTION

Regulation of complex processes like tissue growth control can be mediated by environmental cues, including pH. Intracellular pH (pHi) is regulated by ion transport proteins including the plasma membrane resident sodium-proton exchanger NHE1 (*DNhe2* in *Drosophila melanogaster,* Grillo-Hill *et al.*, 2015). NHE1 exchanges an extracellular Na^+^ for an intracellular H^+^ to maintain pHi near neutral at 7.2. Constitutively increased pHi is a common characteristic of most cancers and is sufficient to induce cancer cell behaviors, including dysplasia, increased proliferation, invasive cell migration, and impeded differentiation (Grillo-Hill *et al.*, 2015; Ulmschneider *et al.*, 2016). Increased pHi is caused by higher expression or activity of ion transport proteins like the ubiquitously expressed sodium-proton exchanger NHE1 (Reshkin *et al.*, 2014). The adult *Drosophila* compound eye is composed of approximately 750 unit eyes, termed ommatidia, that are precisely arranged in a hexagonal lattice (Ready *et al.*, 1976). *Drosophila* eye development is an excellent system to probe how cell signaling pathways coordinate complex biological processes like cell growth and regulated cell death. In the developing *Drosophila* eye, overexpression of *DNhe2* is sufficient to increase pHi, disrupt tissue architectures, increase proliferation, and induce planar cell polarity defects.

Cell number is precisely regulated in the developing *Drosophila* eye in distinct temporal phases for proliferation (larval) and apoptosis (pupal). The eye imaginal disc, which is the precursor to the adult eye, begins as a sheet of undifferentiated cells. During the first and second larval instars, the *Drosophila* eye imaginal disc proliferates and remains largely undifferentiated. During the third larval instar, two bursts of regulated proliferation, termed the first and second mitotic wave, flank the morphogenetic furrow. Posterior to the second mitotic wave, stochastic cell division occurs (Ready *et al.*, 1976; Wolff and Ready, 1991a). At the end of the larval phase of development, proliferation is complete.

Next, apoptosis plays an important role in refining the pattern during pupal eye development in two distinct phases that are regulated by Wnt, Notch, and EGFR signaling (Cagan and Ready, 1989; Yu *et al.*, 2002; Cordero *et al.*, 2004). This process removes exactly seven interommatidial lattice cells from each ommatidium (Wolff and Ready, 1991b). At the end of patterning in midpupal development, this leaves exactly six secondary pigment cells, three tertiary pigment cells, and three bristle complexes surrounding each ommatidium. Regulated cytosolic pH dynamics are observed during apoptosis, with alkalization of mitochondrial matrix pH and subsequent cytosolic acidification. Caspase activation requires decreased pHi, and the highest caspase activity was detected at pH 6.3–6.8 (Matsuyama *et al.*, 2000). Further, one study has shown that constitutively increased pHi blocks apoptotic signaling as measured by downstream effects on DNA degradation (Pérez-Sala *et al.*, 1995). These data led to a model where increased pHi protects cancer cells from apoptotic cell death (White *et al.*, 2017).

Much less is understood about the regulation of necrotic and autophagic cell death in the developing *Drosophila* eye. Different modes of cell death are differentiated by distinct cell morphologies and often initiated through distinct signaling pathways (Ouyang *et al.*, 2012). Necrosis is a form of cell death that is often brought on by injury, which results in the loss of cellular membrane structure and uncontrolled release of cellular contents (Proskuryakov *et al.*, 2003). This initiates an inflammatory response that attracts phagocytes to remove dead cells and leukocytes, which contain microbicidal products to help clear invading microorganisms (Rock and Kono, 2008).

Autophagy is a conserved catabolic process where cellular components are packaged into a double-membrane vesicle known as an autophagosome that fuses with a lysosome to degrade macromolecules. Autophagosome contents are broken down into cellular building blocks (nucleotides, amino acids, and fatty acids) and recycled for homeostasis or growth in a cell autonomous manner (Khandia *et al.*, 2019). In *Drosophila* eye imaginal discs, autophagy is required for tissue differentiation and the survival of precursor cells. Atg5-positive and Atg8a-positive focal labeling was localized within and posterior to the morphogenetic furrow (Billes *et al.*, 2018). Knockdown of *Atg3*, *Atg14*, or *Atg101* via RNA interference (RNAi) results in significantly reduced adult eyes (Billes *et al.*, 2018). Furthermore, when *Atg8a*, *Atg7,* or *Atg9* are knocked down with coexpression of oncogenic *Ras^V12^*, adult eyes show overgrowth or enhanced roughness (Manent *et al.*, 2017).

There are a few examples in development showing that autophagy can culminate in cell death, termed autosis (Das *et al.*, 2012; Liu and Levine, 2015). During metamorphosis in *Drosophila*, larval tissues undergo histolysis in response to the hormone ecdysone. The most studied example is salivary glands; however, their destruction is not solely due to autophagy, as apoptotic caspases are also required for their destruction (Das *et al.*, 2012). However, the amnioserosa cells in the *Drosophila* embryo and midgut cells in *Drosophila* larvae undergo cell death that requires autophagy proteins but does not require caspase activity (Mohseni *et al.*, 2009). In cancer cells, autophagy is often up-regulated and is thought to play a protective, prosurvival role for tumor cells (Debnath *et al.*, 2023). Previous studies have suggested that acidic extracellular pH may regulate autophagy (Wojtkowiak and Gillies, 2012; Zhao *et al.*, 2016), but the mechanism remains unresolved.

We became interested in how dysregulated pHi modulates mechanisms of growth control. We overexpressed *DNhe2* using the eye-specific Glass Multimerized Reporter Gal4 driver (*GMRGAL4*) and found that *GMR>DNhe2* expressing flies showed increased pHi from ∼7.3 to ∼7.7 and induced patterning errors visible as a rough eye phenotype in adult flies (Grillo-Hill *et al.*, 2015). We showed that the rough eye phenotype was due to ion exchange rather than another function of *DNhe2* by expressing a transport dead *DNhe2^E358I^* transgene, which showed no change in pHi and also showed no retinal phenotypes. *GMR>DNhe2* expressing flies showed an increase in stochastic proliferation in the posterior region of the eye imaginal disk (Grillo-Hill *et al.*, 2015). With increased proliferation, we expected to see an increase in the size of the adult eye; however, external examination of adult fly eyes revealed an overall size decrease. Here, we investigate the cellular basis of this reduced eye size, and report that *DNhe2* overexpression induces a 20% decrease in the number of interommatidial cells in the developing eye. In addition, we found that mutations in essential apoptotic genes including the viral caspase inhibitor *p35* show no effect on cell loss. However, decreasing activity of autophagy genes suppressed the reduced eye phenotype and rescued missing cells. Further, molecular markers support increased autophagy. Collectively, our data suggest that regulated autophagy occurs in response to increased pHi, and suggests a model for regulation of autophagy in tumors where autophagic cell death may promote tumor growth.

## RESULTS AND DISCUSSION

### Overexpression of *DNhe2* causes a smaller adult eye and loss of interommatidial lattice cells

**FIGURE 1:**
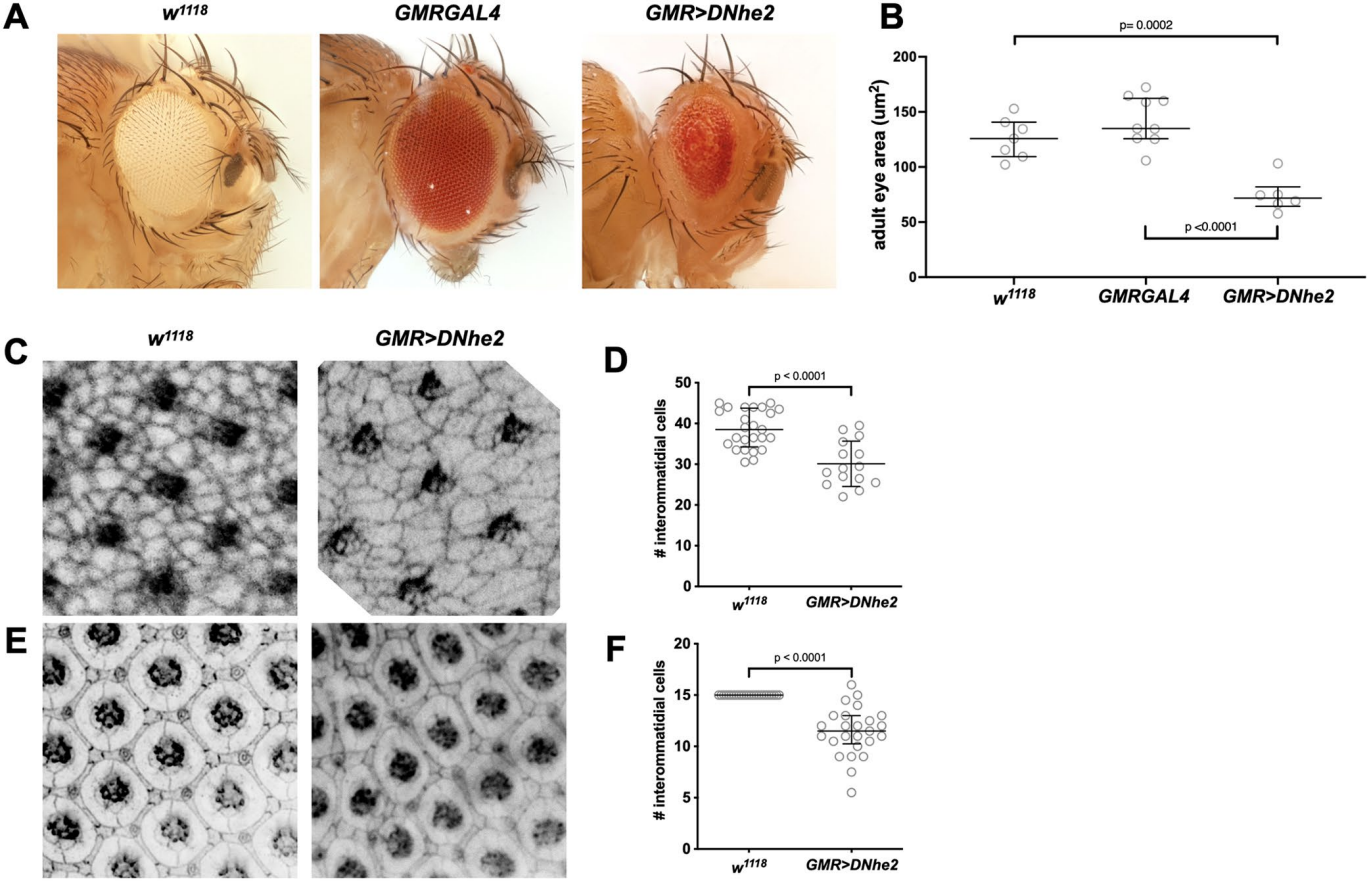
Overexpression of *DNhe2* induces a rough, small eye and reduces interommatidial cell number. (A) High-resolution images show adult eyes for these genotypes: *w^1118^* (parental strain control); *GMRGAL4* heterozygotes (driver control); *GMR>DNhe2*. (B) Mean measured adult eye sizes were: *w^1118^* (125.83 µm^2^, *N* = 7); *GMRGAL4* (142.49 µm^2^, *N* = 9), and *GMR>DNhe2* (74.28 µm^2^, *N* = 6). One eye per adult fly was imaged and quantified. Data are graphed as median values with interquartile ranges. Statistical significance was determined by unpaired *t* tests with Welch’s correction. (C) Confocal micrographs show *w^1118^* and *GMR>DNhe2* wandering third larval instar eye imaginal discs labeled with an antibody against Armadillo (Arm, *Drosophila* beta-catenin) to highlight cell-cell junctions. (D) Mean cell counts were: *w^1118^*(38.6 cells, *N* = 25 counting areas from 5 discs/animals); *GMR>DNhe2* (30.10 cells, *N* = 15 counting areas from 3 discs/animals). Data are graphed as median values with interquartile ranges. Statistical significance was determined by unpaired *t* tests with Welch’s correction. (E) Confocal micrographs show *w^1118^* and *GMR>DNhe2* 32H apf pupal eyes labeled with an antibody against Armadillo (Arm) to highlight cell-cell junctions. (F) Mean cell counts were: *w^1118^* (15 cells, *N* = 25 counting areas from 5 eyes/animals); *GMR>DNhe2* (11.42, *N* = 25 from 5 eyes/animals). Data are graphed as median values with interquartile ranges. Statistical significance was determined by unpaired *t* tests with Welch’s correction.

To determine how increased *DNhe2* expression impacts tissue size in the *Drosophila* eye, quantitative analysis of the area of adult eyes was performed. High-resolution images of adult *Drosophila* eyes were collected (Figure 1A), and the area of each eye was measured. We found that *GMR>DNhe2*-expressing flies have significantly smaller eyes (mean 74.28 µm^2^) compared with the genetic background control *w*^*1118*^ flies (mean 125.83 µm^2^) or the driver control *GMRGAL4* heterozygous flies (mean 142.49 µm^2^), respectively 41% and 48% reduction in area (Figure 1B). This reduction in eye size, despite increased proliferation, suggests that cell death may be increased.

To determine when in development this decrease occurs, cell counts of interommatidial lattice cells were performed in wandering third larval eye imaginal discs and in pupal eyes at the end of patterning. Cell junctions were labeled and cells were counted through identification of a central ommatidium and its six nearest neighbors. Counting areas defined by lines connecting through centers of neighboring ommatidium. Cells completely contained within this hexagon count as one cell, and partially contained cells count as one-half cell. In *w*^*1118*^ wandering third larval eye imaginal discs, we found an average of 38.6 cells per counting area, compared with 30.1 cells in *GMR>DNhe2* (Figure 1, C and D), a reduction of ∼22%. In pupal eyes, we counted interommatidial lattice cells and bristles at the end of pattern formation, and found a decrease from exactly 15 cells per counting area in control to 11.4 cells per counting area in *GMR>DNhe2* expressing pupal eyes (Figure 1, E and F), a reduction of ∼24%. These data suggest that cell loss is apparent during larval development, and that these missing cells are not restored by the end of pattern formation in pupae.

**FIGURE 2:**
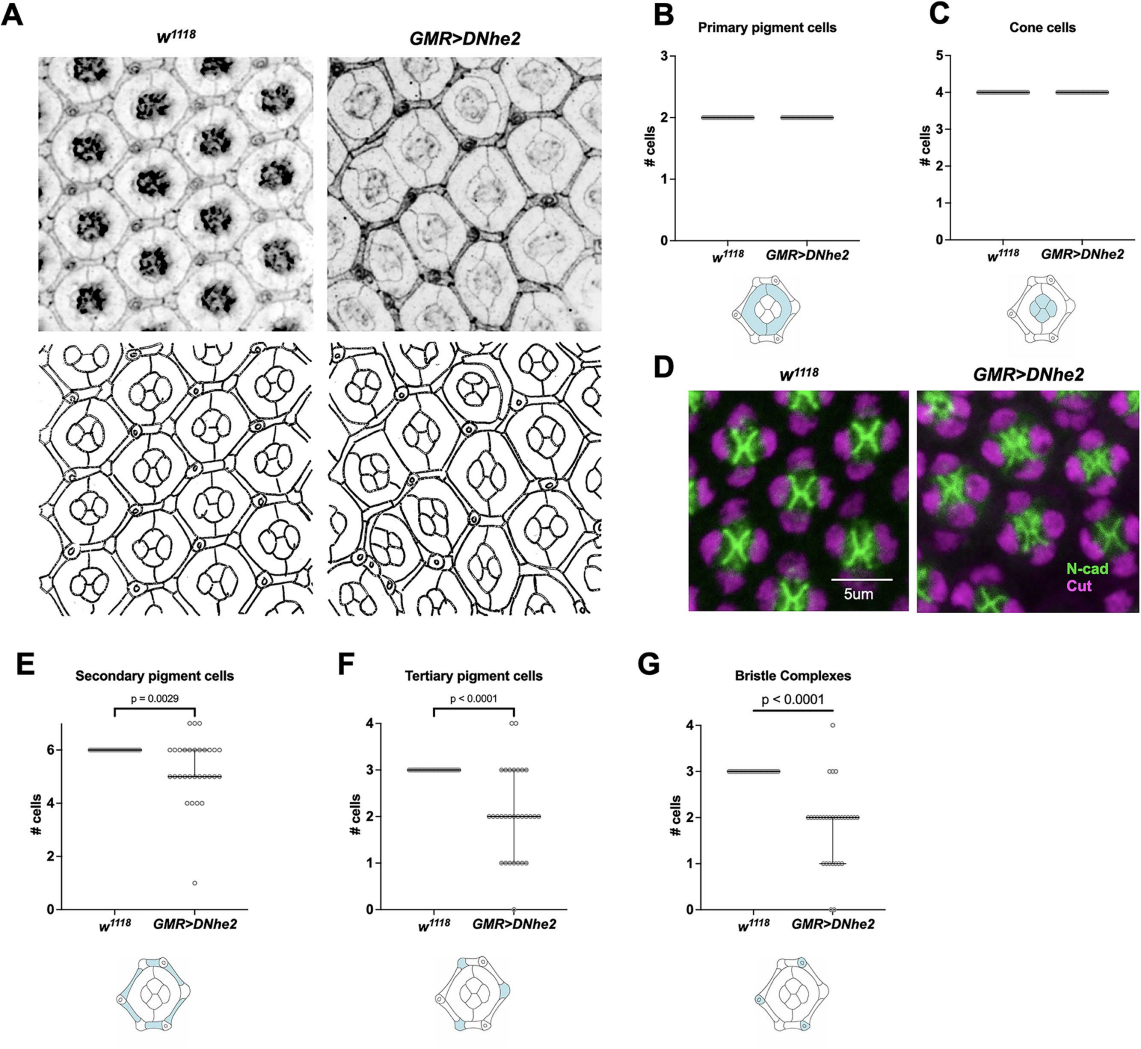
Decreased numbers of secondary pigment cells, tertiary pigment cells, and bristle complexes with overexpression of *DNhe2*. (A) Pupal retinae immunolabeled for Armadillo (Arm, *Drosophila* beta-catenin) to highlight cell-cell junctions in *w^1118^* and *GMR>DNhe2* flies. Schematic diagrams (below) are tracings of micrographs. (B) Cell counts of primary pigment cells (highlighted in blue) in *w^1118^* and *GMR>DNhe2*. (C) Cell counts of cone cells (highlighted in blue) in *w^1118^* and *GMR>DNhe2*. (D) Expression of cone cell markers N-cadherin (N-cad, green) and Cut (magenta) in *w^1118^* and *GMR>DNhe2*. (E) Secondary pigment cell counts (highlighted in blue) in *w^1118^* (mean count = 6) and *GMR>DNhe2* (mean count = 5.3). (F) Tertiary pigment counts (highlighted in blue) in *w^1118^* (mean count = 6) and *GMR>DNhe2* (mean count = 2). (G) Bristle complex counts (highlighted in blue) in *w^1118^*(mean count = 3) and *GMR>DNhe2* (mean count = 1.8). Data are graphed as median values with interquartile ranges. Statistical significance was determined by unpaired *t* tests with Welch’s correction; *N* = 30 ommatidia from 5 individual flies for both genotypes.

As previously reported, ommatidial organization in *GMR>DNhe2* is significantly disrupted compared with control *w^1118^*, specifically cells show aberrant cell-cell contacts, and aberrant cell numbers when cell-cell junctions are labeled with an antibody against Armadillo, the *Drosophila* ortholog of beta-catenin (Figure 2A). Specific defects included missing secondary and tertiary pigment cells, doubled secondary pigment cells, and asters (four pigment cells surrounding bristles at ommatidial vertices). To quantify these observations, we performed cell counts for each non-neuronal cell type visible on the apical surface of the retinal epithelium in pupal eyes: cone cells, primary pigment cells, secondary pigment cells, tertiary pigment cells, and bristles. We did not observe a change in the number of primary pigment cells (Figure 2B) or cone cells (Figure 2C). Cone cell markers N-cadherin and Cut were expressed in the normal pattern (Figure 2D). Secondary pigment cells decreased from 6 cells per ommatidium to 5.3 cells per ommatidium, ∼12% reduction (Figure 2E). Tertiary cells decreased from 3 cells per ommatidium to 2.1 cells per ommatidium, ∼30% reduction (Figure 2F). Finally, bristle complexes decreased from 3 per ommatidium to 1.8 bristle complexes per ommatidium, which is ∼40% reduction (Figure 2G). This decrease in cell number during development corresponds to the smaller appearance of adult eyes and could reflect decreased proliferation or increased cell death. We previously published that *GMR>DNhe2* expression increases stochastic proliferation in eye imaginal discs (Grillo-Hill *et al.*, 2015), so we hypothesized that reduced cell number results from increased cell death.

### Genetic interaction studies support *DNhe2*-induced autophagic cell death

We next investigated which mode of cell death (necrosis, apoptosis, or autophagy) removes cells with *GMR>DNhe2* expression. Necrotic tissue in the *Drosophila* eye shows disrupted membranes and a lack of architecture in histological sections. We ruled out necrosis as our previous histological analysis of adult retinae in *GMR>DNhe2* flies found that cell bodies are intact, and membrane-rich rhabdomeres are present and correctly patterned, allowing for the previously described planar cell polarity defects (Simons *et al.*, 2009; Grillo-Hill *et al.*, 2015). This suggests that apoptosis or autophagy is responsible for the missing cells. The secondary pigment cells, tertiary pigment cells, and bristle complexes cell types are collectively called interommatidial cells and are known to be selectively eliminated through apoptosis in larval and pupal development.

**FIGURE 3:**
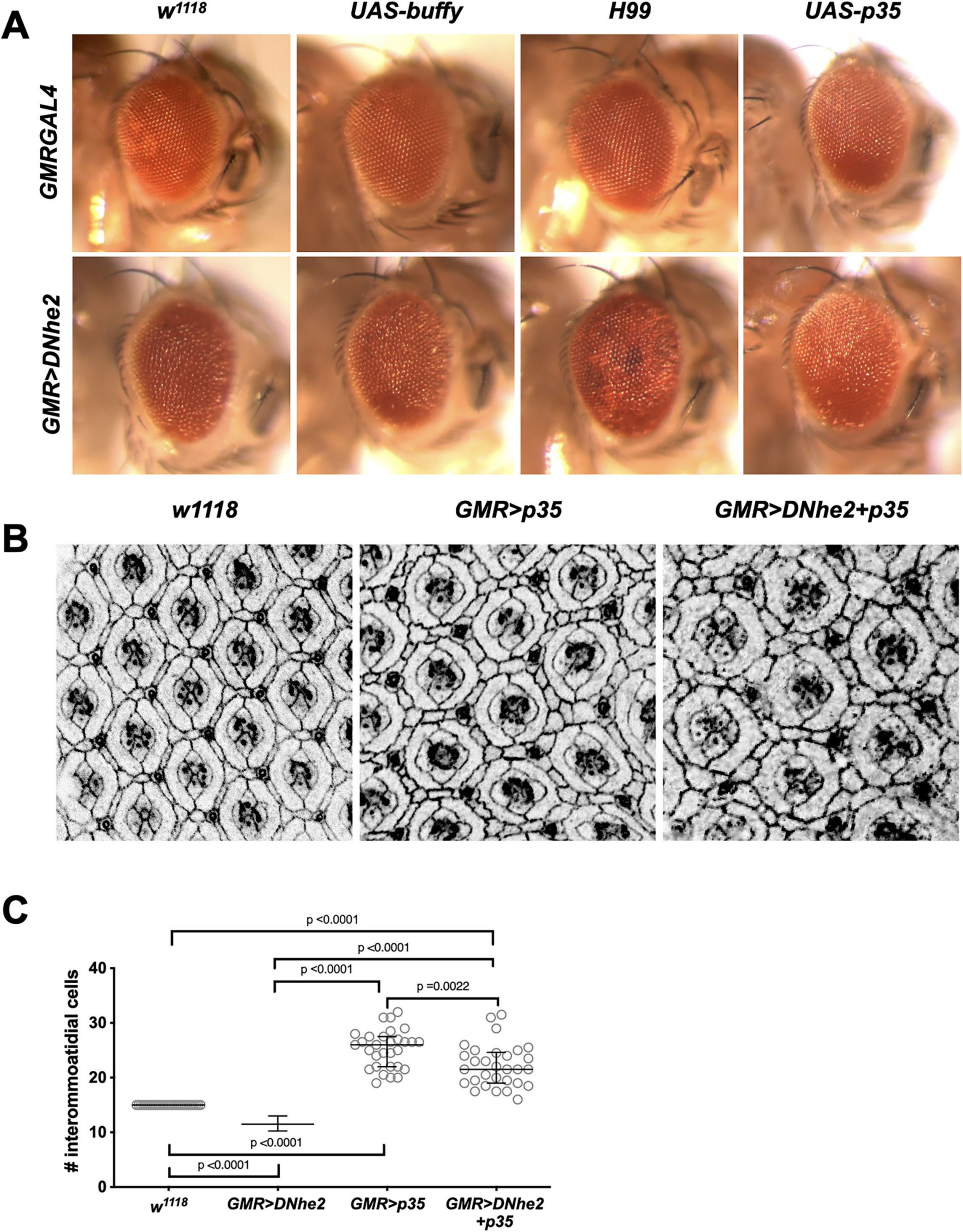
Caspase inhibition does not completely restore missing interommatidial cells. (A) Light micrographs showing adult eyes from the following genotypes (left to right): top row: *GMRGAL4; GMR>buffy*; *GMRGAL4*; *H99; GMR>p35;* bottom row: *GMR>DNhe2*; *GMR>DNhe2*+*UAS-buffy*; *GMR>DNhe2*; *H99*; *GMR>DNhe2*+*UAS-p35*. (B) Confocal micrographs of mid-pupal eyes immunolabeled for Arm to show cell-cell junctions in control (*w^11^^18^*), *GMR>p35* alone, and *GMR>DNhe2+p35*. (C) Quantification of interommatidial cell number in *w^1118^* (mean = 15 cells), *GMR>DNhe2* (mean = 11.4 cells), *GMR>p35* (mean = 25.3 cells), and *GMR>DNhe2+p35* (mean 22.2 cells). Data are graphed as median values with interquartile ranges. Statistical significance was determined by unpaired *t* tests with Welch’s correction; *N* = 30 ommatidia from 6 individual flies for all genotypes except *GMR>DNhe2* (25 ommatidia from 5 flies). Cell count data for *GMR>DNhe2* are from data shown in Figure 1F.

We predicted that if apoptosis is responsible for the reduction in cell number, then inhibiting apoptosis should rescue the *GMR>DNhe2* rough eyes, restoring normal patterning and missing cells. We tested for genetic interactions with anti-apoptotic genes, including *buffy*, *H99* deletion and overexpression of the viral caspase inhibitor *p35* (Figure 3A). Previous studies show that overexpression of the anti-apoptotic gene *buffy* produces flies with wild-type eyes and blocks caspase-dependent cell death (Quinn *et al.*, 2003). Overexpression of *buffy* alone using the *GMRGAL4* driver showed no effect on retinal patterning and did not show a genetic interaction with *GMR>DNhe2* (Figure 3A). The *H99* deletion removes three genes, *grim*, *reaper* (*rpr)*, and *head involution defective (hid)* (White *et al.*, 1994). The genes *grim*, *rpr,* and *hid* inhibit the inhibitor of apoptosis proteins (IAP) proteins, which in turn inhibit caspases, hence the *H99* deficiency indirectly blocks apoptotic cell death via IAP inhibition of caspases (Yu *et al.*, 2002). *H99* heterozygous flies do not display visible patterning errors. *GMR>DNhe2* flies that were heterozygous for *H99* showed a moderate suppression of the reduced eye size but retained patterning defects (Figure 3A). Finally, we tested *p35*, a viral caspase inhibitor and anti-apoptotic protein that completely inhibits apoptosis (Hay *et al.*, 1994). Expression of *p35* alone appeared wild-type, and when *p35* and *DNhe2* were coexpressed, the rough eye phenotype was suppressed (Figure 3A), causing the adult eye to appear larger and more organized. We saw this suppression consistently using three different *p35* transgenic fly lines.

We predicted that if apoptosis is the pH-sensitive mode of cell death, then inhibiting caspases should restore the missing cells observed in *GMR>DNhe2* pupal eyes. We quantified cell number with *p35* expression alone, or with *GMR>DNhe2*. In pupal eyes, expression of *p35* alone did not disrupt patterning (Figure 3B, middle), and increased the number of interommatidial cells from 15 in control to 25.3, suggesting that normally ∼10 cells are removed through apoptosis (Figure 3, B and C). Coexpression of *p35* and *DNhe2* (Figure 3B, right) increased cell number to 22.2, compared with 11.4 in *GMR>DNhe2* alone, which is a difference of ∼11 cells. These data show inhibition of caspases through expression of *p35* does not completely rescue the missing cells induced by overexpression of *DNhe2*, as there are ∼3 fewer cells in *GMR>DNhe2+p35* (22.2 cells) compared with *GMR>p35* alone (25.3 cells). These data suggest that apoptosis does not cause the reduction in cell number with *GMR>DNhe2* expression, since coexpression of *p35* did not revert cell number to wild-type levels.

### Autophagic cell death is increased with *DNhe2* overexpression

**FIGURE 4:**
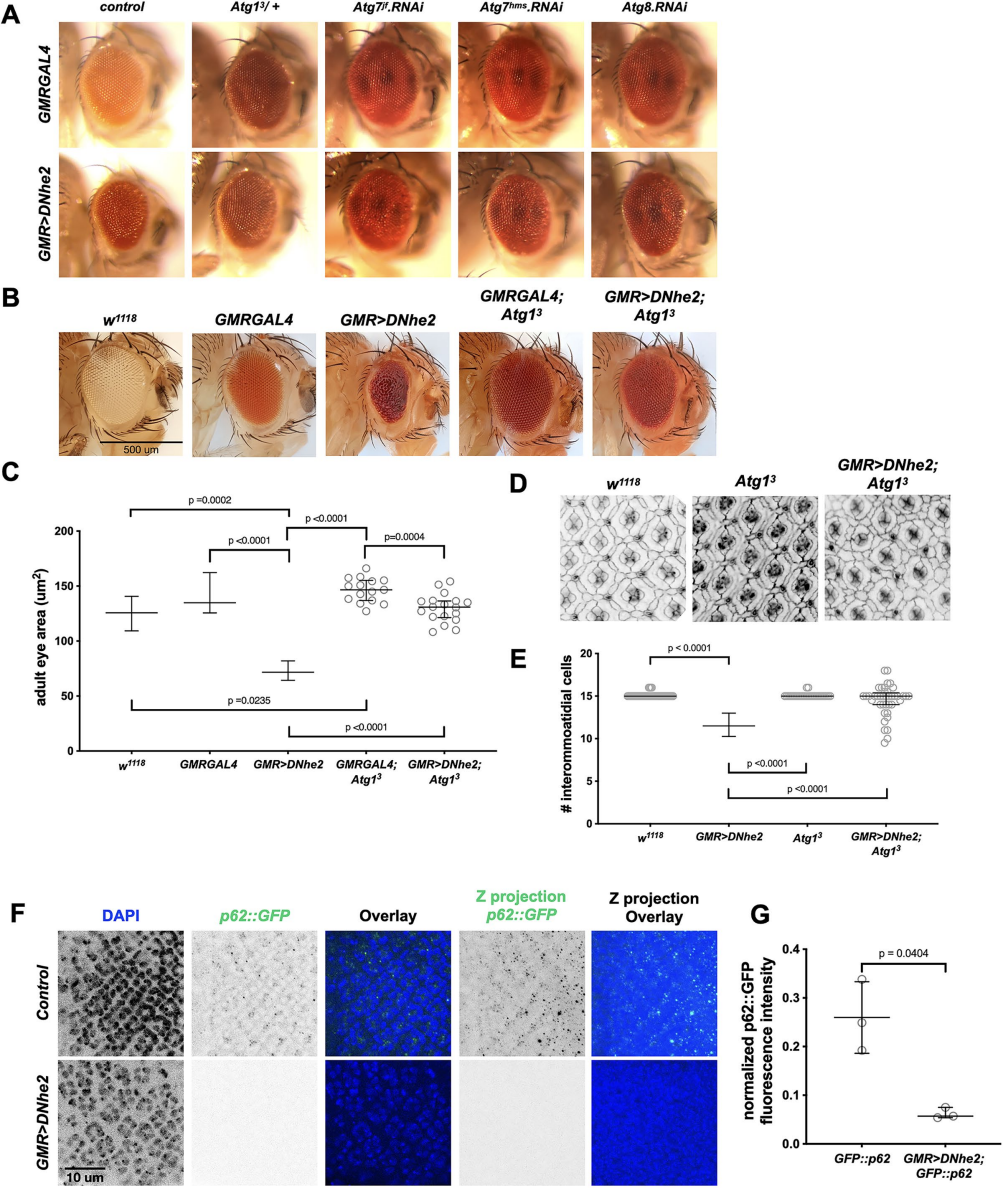
Genetic interactions, phenotypic suppression, and marker analysis support autophagic cell death with *GMR>DNhe2* expression. (A) Light micrographs showing adult eyes from the indicated genotypes. (B) High-resolution images of adult fly eyes used for eye size quantification for the following genotypes: *w^1118^*; *GMRGAL4*; *GMR>DNhe2*; *GMRGAL4*; *Atg1^3^*; *GMR>DNhe; Atg1^3^*. (C) Mean measured adult eye sizes were shown in Figure 1 for *w^1118^*(125.83 µm^2^, *N* = 7); *GMRGAL4* (142.49 µm^2^, *N* = 9), and *GMR>DNhe2* (74.28 µm^2^, *N* = 6), and indicated by showing median values with interquartile ranges. Mean measured adult eye sizes were *GMRGAL4; Atg1^3^* (146.4 µm^2^, *N* = 15); *GMR>DNhe2; Atg1^3^* (129.9 µm^2^, *N* = 19). (D) Confocal micrographs of 32H apf pupal eyes of the indicated genotypes, labeled with Armadillo to outline cells. (E) Quantification of interommatidial cell number of the indicated genotypes. Mean values from Figure 1F were: *GMR>DNhe2* (mean = 11.42 cells, *N* = 25 counting areas from 5 flies). Measured mean values were: *w^1118^*(15.1 cells, *N* = 51 counting areas from 11 flies); *Atg1^3^* (14.5 cells, *N* = 30 counting areas from 8 flies); *GMR>DNhe2; Atg1^3^* (15.1 cells, *N* = 30 counting areas from 8 flies). (F) Confocal micrographs of wandering third larval eye imaginal discs expressing *GFP::p62* from control or *GMR>DNhe2*-expressing flies. Panels show single-channel images for DAPI (blue), *GFP::p62* (green), an overlay, and a maximum projection through the entire tissue. (G) Quantification of *GFP::p62* intensity from control or *GMR>DNhe2*-expressing flies. Data are graphed as median values with interquartile ranges. Statistical significance was determined by unpaired *t* tests with Welch’s correction; *N* = 3 individual flies (3 Z-slices averaged per fly).

We performed genetic interaction studies with known autophagy genes (Figure 4A)*.* We tested mutations in several genes that facilitate autophagy: *Atg1,* which induces phagophore formation; *Atg7* which facilitates vesicle completion; and *Atg8a* which regulates autophagosome maturation. The *Atg1^3^* loss-of-function allele showed no effect on eye development alone, but phenotypically suppressed the *GMR>DNhe2* rough eye phenotype (Figure 4A). We decreased expression levels of *Atg7* and *Atg8a* via RNAi, which showed similar effects. These interactions suggest autophagy may be enhanced with *GMR>DNhe2* expression. We quantified effects on adult eye size using high-resolution photography and analysis of area (Figure 4B). We found that *GMR>DNhe2* flies heterozygous for *Atg1^3^* showed a restoration of eye size (129.9 µm^2^, compared with 74.28 µm^2^ for *GMR>DNhe2* alone shown in Figure 1), and were indistinguishable from their respective controls (Figure 4C).

To quantify the effects on cell number, we counted interommatidial cells at the end of pattern formation in pupal eyes in flies heterozygous for *Atg1^3^* alone, or with expression of *GMR>DNhe2*. Flies heterozygous for *Atg1^3^* showed no defects in patterning or cell counts compared with *w^1118^* control flies. Flies expressing *GMR>DNhe2* and heterozygous for *Atg1^3^* appeared rescued with respect to cell number; however, some patterning errors in interommatidial cells and cone cells were evident (Figure 4D). Flies heterozygous for *Atg1^3^* are comparable to *w^1118^* control flies. Flies heterozygous for *Atg1^3^* and expressing *GMR>DNhe2* showed a nearly complete rescue of cell number, with an average cell count of 14.5 compared with 11.42 with *GMR>DNhe2* alone, and 15 in control *w^1118^* flies (Figure 4E).

To determine whether autophagy levels are increased, we used a ubiquitously-expressed p62 protein with an N-terminal-GFP tag (Kiss *et al.*, 2020) alone or with *GMR>DNhe2*. p62 is an adaptor protein that recruits proteins targeted for lysosomal degradation, and accumulates when autophagy is inhibited or decreases when autophagy is induced (Bjørkøy *et al.*, 2009, Chapter 12). p62 has proven to be a reliable marker for autophagy (Filimonenko *et al.*, 2007). Wandering third larval instar eye imaginal discs were dissected, fixed, and labeled, and confocal images were collected of tissues expressing *GFP::p62* alone or with *GMR>DNhe2*. *GFP::p62* labeling is visible as distinct puncta throughout the third larval instar eye imaginal tissue, both in single Z-slices as well as in a Z-stack projection throughout the entire tissue (Figure 4F). We observed a significant decrease in *GFP::p62* fluorescence intensity with expression of *GMR>DNhe2* compared with *w^1118^* control, with a normalized mean integrated density values of 0.2597 compared with 0.6200 in *w^1118^* control (Figure 4G). Together, these data strongly support increased autophagic cell death with *GMR>DNhe2* expression.

## CLOSING THOUGHTS

Our research has shown for the first time that overexpression of *DNhe2* results in reduced cell number, likely through autophagic cell death. Our established *Drosophila* models overexpress the sodium-proton exchanger *DNhe2* in the developing eye, which results in increased pHi and a rough, disorganized, smaller eye in adult flies. We showed reduced interommatidial cell number with overexpression of *DNhe2*. Genetic interaction studies with apoptotic genes showed incomplete phenotypic suppression and did not rescue cell number. However, genetic interactions with autophagy genes suppressed the *GMR>DNhe2* rough eye phenotype and rescued missing cells. Finally, expression of molecular markers supports increased autophagy with increased *DNhe2* expression. These data support a new model where increased pHi is sufficient to induce autophagic cell death, which has implications for basic biological functions such as proteostasis, and for diseases with dysregulated autophagy like cancer.

One interesting consequence of our studies is the unraveling of pHi-regulated cellular processes during morphogenesis of the developing *Drosophila* eye. Our genetic interaction studies previously identified pH-regulated proteins like beta-catenin, where coexpression of the *Drosophila* orthologue *Armadillo* showed complete phenotypic suppression of the *GMR>DNhe2*-induced rough eye phenotype. In this study, we observed that distinct aspects of the *GMR>DNhe2* phenotype can be genetically dissected, as removing one copy of *Atg^1^* results in phenotypic suppression and restoration of the missing interommatidial cells. However, patterning errors are still visible in the pupal eyes, suggesting abnormalities in cone cell differentiation or survival and in planar cell polarity. These defects contrast with phenotypes seen with coexpression of *p35*, where extra interommatidial cells are present but have a minimal impact on tissue patterning due to reduced apical cell profiles. This supports the idea that increased pHi impacts different signaling pathways during *Drosophila* eye development, and that these effects can be disaggregated through genetic interaction studies and subsequently characterized through cell biological studies.

Finally, our data shed insights into the burgeoning field of autophagy research. Many studies in the autophagy field currently focus on specific protein or organelle targets that are degraded through autophagy. It is not known how these findings interface with the less-studied phenomenon of autophagic cell death, also called autosis; the classification of autophagy as a regulated mode of cell death is itself still controversial (Liu and Levine, 2015; Nah *et al.*, 2020; Bai *et al.*, 2023). While the same proteins and biological processes are required for these processes, it is unclear whether they represent a continuum, or distinct processes. Homeostatic autophagy may become increased or dysregulated beyond a threshold, resulting in autophagic cell death. Alternately, distinct signaling cues or signal strength modulation upstream of autophagosome formation may impact the ultimate outcome of surviving cellular stresses or induction of cell death. To further complicate matters, there are complex interconnections between autophagy proteins and induction of cell death through apoptotic or necrotic pathways (Maiuri *et al.*, 2007; Nikoletopoulou *et al.*, 2013; Saleem, 2021; Sorice, 2022). Hence, our model showing increased autophagy at higher pHi in the developing *Drosophila* eye may lend novel insight in these questions, especially as the *Drosophila* eye was a significant model system in elucidating apoptotic signaling.

Autophagy is known to be increased in cancer. Overall rates of cell death are decreased in tumors, which is thought to be partially due to inhibition of acid-activated caspases, preventing apoptosis (White *et al.*, 2017). We hypothesize that higher pHi common to cancer cells induces autophagy to permit cells that are stressed or dying from metabolic dysfunction, oxidative stresses, or inflammation to fuel tumor growth by providing building blocks to the surrounding, healthier tumor cells.

## MATERIALS AND METHODS

Request a protocol through *Bio-protocol*.

### Drosophila stocks

Flies were maintained on Nutri-Fly Bloomington Formulation media (Genesee, catalogue no. 66-113) and maintained at 25°C unless otherwise stated. *Drosophila* stocks were obtained from the Bloomington Drosophila Stock Center (Bloomington, IN): *w^1118^* (#3605); *w; Kr/CyO, mef2>CD8::RFP* (#26882Caspase inhibitor fly strains: w*; P{w[+mC] = UAS-p35.H}BH1 (*UAS-p35-1*, #5072); w*;P{w^+mC^= UAS-p35.H}BH2 (*UAS-p35-2*, #5073); w*; P{w^+mC^= GMR-p35}X-1, and (*GMR>p35*, #5774); *Df(3L)H99, kni^ri-1^ p^p^/TM3, Sb^1^* (*H99*, #1576); w*; P{w^+mC^= UAS-Buffy.S}E1 (*UAS-Buffy*, #32059); w*; Atg1^3^ P{ry^+t7.2^= neoFRT}82B/TM3, Sb^1^ Ser^1^ (*Atg1^3^*, #60732); y^1^ sc* v^1^ sev^21^; P{y^+t7.7^ v^+t.^^1.8^= TRiP.HMS01358}attP2/TM3, Sb^1^ (*Atg7^hms^.RNAi*, #34369); *y^1^ v^1^; P{y^+t7.7^v^+t1.8^= TRiP.JF02787}attP2* (*Atg7^jf^.RNAi*, #27707); y^1^ sc* v^1^ sev^21^; P{y^+t7.7^ v^+t1.8^= TRiP.HMS01328}attP2 (*Atg8a.RNAi*, #34340). Additional stocks used: *w;GMRGAL4* (outcrossed to a single copy when used as a control; gift from R. Cagan,); *tub>GFP::p62 3.7M (GFP::p62*; gift from G. Juhasz); *w^1118^*; *GMR>DNhe2*/CyO (Grillo-Hill *et al.*, 2015).

### Genetic interaction studies

Genetic interaction studies were performed in duplicate or triplicate sets of crosses. P0 flies were discarded when F1 larvae were visible. Adult eye phenotypes were documented using a stereomicroscope equipped with a Zeiss AxioCam MRc5 camera using Micro-Manager to acquire images and FIJI (NIH Image J, National Institutes of Health, Bethesda, MD) to adjust display settings.

### High-resolution images of adult *Drosophila* eyes

Flies were generated through genetic crosses or taken from stocks, and the appropriate genotypes were selected by phenotypic markers. Flies were anesthetized with CO_2_ and placed into 70% ethanol in microcentrifuge tubes. Flies were imaged within 6 h, or stored at 4°C and imaged within 24 h. Flies were prepared for imaging by point mounting. Briefly, flies were glued laterally to the tip of a small piece of triangular cardstock, and pinned with a #2 or #3 entomological pin. For each pinned specimen, one eye was imaged. Images were acquired on a Canon EOS 6D Mark II camera, with a Canon EF 70-200 mm USM II telephoto lens with an attached Mitutoyo 20X Apo Microscope Objective. Focus stacked images of each specimen were produced using a Stackshot Controller and Rail system.

Adult eye size was quantified using ImageJ (National Institutes of Health, Bethesda, MD). An outline of the adult fly eye was made with the freehand selection tool. In the “Analyze” menu, we selected “Measure” to quantify area, mean, minimum, and maximum values. Statistical analyses were performed using Prism (Graphpad Software). Outliers were removed and statistical analysis used one-way ANOVA or Welch’s *t* test. Scaling was performed according to the imaging software, where 1 pixel = 3016 mm.

### Larval and pupal eye dissection, fixation, and imaging

White prepupae were transferred into a humidified plastic dish and incubated at 25°C for 32 h after puparium formation (32H apf). Wandering third instar eye imaginal discs or 32H apf pupal eyes were dissected in PBS then fixed in fresh 4% paraformaldehyde (catalogue no. 15710, Electron Microscopy Sciences, Hatfield, PA) in PBS for 20 min, washed three times 10 min in PBT (PBS + 0.1% Triton X-100 [catalogue no. BP151-500 Sigma-Aldrich, St. Louis, MO]), and incubated at 4°C overnight with mouse anti-Armadillo antibody 1:10 (catalogue no. N2 7A1, Developmental Studies Hybridoma Bank, Iowa City, IO) in PBT + 5% normal goat serum (catalogue no. 642921, MP Biomedicals, Solon, OH). The next day, tissues were washed three times 10 min in PBT, incubated in goat anti-mouse AlexaFluor Plus 555 1:500 (catalogue no. A32727, Thermo Fisher Scientific) and Hoechst 33342 (1:10,000; catalogue no. H3570; Invitrogen, Thermo Fisher Scientific) for 2 h at room temperature in the dark, then washed three times 10 min in PBT, mounted in Prolong Gold or Prolong Glass Mountant (catalogue no. P10144 or no. P36961, Molecular Probes by Life Technologies) and cured overnight at room temperature in the dark then transferred to −20°C until imaging.

Image stacks were collected on a Zeiss LSM 700 confocal microscope using a 40x oil immersion objective (420462-9900-000, Zeiss Objective EC Plan-Neofluar 40x/1.30 Oil DIC M27); *p62::GFP* imaging or 63x oil immersion objective (420782-9900-000, Zeiss Objective Plan-Apochromat 63x/1.4 Oil DIC M27). High-resolution images (2048 × 2048) were acquired using the z-stack function (ZEN Software) on a high-resolution AxioCam microscope camera. Within each set of experiments, images were acquired using identical settings enabled by the “reuse settings” feature of the ZEN software. Images were adjusted and analyzed using FIJI Software (National Institutes of Health, Bethesda, MD). Control and experimental conditions were imaged on the same day.

#### p62::GFP imaging.

Within each set of experiments, images were acquired using identical settings enabled by the “reuse settings” feature of the ZEN software. The following pixel parameters were measured using FIJI in all image slices in all channels: mean, min, max, integrated density, and median. The slice with the highest integrated density for the Hoechst 33342 label was selected for analysis as this indicates a focal plane with the greatest amount of tissue. The slices immediately above and below were also quantified for technical replicates. The raw integrated intensity value for each channel was also averaged with their two neighboring slice raw integrated values. The GFP channel raw integrated mean intensity value was then normalized to the Hoechst 33342 channel raw integrated mean intensity value to control for tissue area in each slice. Control *(tub>GFP::p62 3.7M)* normalized values were then compared with the normalized values from the experimental group (*w; GMRGAL4, UAS-DNhe2L4 A/tub GFP::p62).* Display settings and crop margins for images in figures were identical for both genotypes.

#### Cell counts.

Cell counts were performed by identifying a central ommatidium and its six nearest neighbors, and drawing a hexagon through the center of the cone cells in the six neighbors. Each cell completely contained in this hexagon counts as one cell, and cells that are partially contained count as one-half cell. Cell counts were performed by at least 3 different people for reproducibility, and any discrepancies were discussed to consensus. One individual eye imaginal disk or pupal eye are taken from each animal.

## Abbreviations used:

apf: after puparium formation
Atg: autophagy-related
*DNhe2*: *Drosophila sodium proton exchanger 2* (Na-H Exchanger)
GFP: green fluorescent protein
*GMRGAL4*: *Glass-multimerized reporter Gal4*
NHE1: sodium proton exchanger 1 (Na-H Exchanger)
RNAi: RNA interference
