# Supplementary material for: Drosophila Nhe2 overexpression induces autophagic cell death
Source: Mol Biol Cell. 2024 Jul 1;35(7):br13. doi: 10.1091/mbc.E24-02-0058 (PMC11244158; doi:10.1091/mbc.E24-02-0058)
Supplement: Supplementary file 1 [file reviewer_reportE24-02-0058.pdf]

Drosophila Nhe2 Over-expression Induces Autophagic Cell Death

Bree Grillo-Hill, Jobelle Peralta, Blake DuPriest, Daniel Orozco, Juan Reyna Pacheco, Laura Martins, Rachel Ann Soriano, Alan Wong, and Ramy Wong

Corresponding author(s): Bree Grillo-Hill, San José State University

Review Timeline:

Submission Date:	2024-02-05
Editorial Decision:	2024-04-04
Revision Received:	2024-04-19
Accepted:	2024-04-21

Editor-in-Chief: Matthew Welch

Transaction Report:

(Note: With the exception of the correction of typographical or spelling errors that could be a source of ambiguity, letters and reports are not edited. The original formatting of letters and referee reports may not be reflected in this compilation.)

RE: Manuscript #E24-02-0058
TITLE: "Increased Intracellular pH is Sufficient to Induce Autophagic Cell Death"

Dear Dr. Grillo-Hill:

Your manuscript "Increased Intracellular pH is Sufficient to Induce Autophagic Cell Death" has been reviewed by two expert referees; their verbatim comments are attached below. Their opinions differed somewhat. Reviewer #2 was mostly enthusiastic and requested only clarification of whether Dnhe2 is indeed homologous to the mammalina NHE1 or instead to NHE3, as claimed by others. This reviewer also made a few comparatively minor suggestions for improvement.
Reviewer #1 was considerably more critical, emphasizing the fact that, contrary to what is implied in the title and elsewhere in the paper, you failed to demonstrate that alkalinization is in fact directly responsible and sufficient to induce autophagic death. As pointed out, this would require manipulation of the pH to either prevent the alkalinization induced by Dnhe2, or induction of a comparable alkalinization by independent means. Reviewer #1 also noted that no pH measurements were reported in this manuscript. Moreover, this reviewer was critical of the earlier pH measurements and had concerns about the extrapolation required to reach the pH values reported.
I feel that you have two possible courses of action: 1) perform and include the suggested experiments in order to justify the claims (and current title) of the manuscript, which would require inclusion of properly calibrated pH determination experiments and separate manipulation of the pH or 2) modify the manuscript to attribute the observed effects to overexpression of Dnhe2, without specifying definitively that alkalinization is responsible for the effects. You could speculate about the possible mechanisms involved, which could presumably include elevated intracellular Na also, without stating definitively that pH changes are in fact the proven mechanism,

Sincerely,
Sergio Grinstein
Monitoring Editor
Molecular Biology of the Cell

Dear Dr. Grillo-Hill,

The review of your manuscript, referenced above, is now complete. The Monitoring Editor has decided that your manuscript requires minor revisions before it can be published in Molecular Biology of the Cell, as described in the Monitoring Editor's decision letter above and the reviewer comments (if any) below.

A reminder: Please do not contact the Monitoring Editor directly regarding your manuscript. If you have any questions regarding the review process or the decision, please contact the MBoC Editorial Office (mboc@ascb.org).

When submitting your revision include a rebuttal letter that details, point-by-point, how the Monitoring Editor's and reviewers' comments have been addressed. (The file type for this letter must be "rebuttal letter"; do not include your response to the Monitoring Editor and reviewers in a "cover letter.") Please bear in mind that your rebuttal letter will be published with your paper if it is accepted, unless you have opted out of publishing the review history.

Authors are allowed 15 days to submit a revision. If this time period is inadequate, please contact us immediately at mboc@ascb.org.

In preparing your revised manuscript, please follow the instruction in the Information for Authors (www.molbiolcell.org/info-for-authors). In particular, to prepare for the possible acceptance of your revised manuscript, submit final, publication-quality figures with your revision as described.

If your manuscript contains a Significance Statement, please make sure that it consists of three separate bullet points totaling a maximum of 100 words and addressing each of the following points (see https://www.molbiolcell.org/curation-tools):
First bullet: What is the background context? What gap in knowledge does this study addres?
Second bullet: What are the key findings? What is unique or new about the approach?
Third bullet: Why is this paper significant? How might it influence future research?

To submit the rebuttal letter, revised version, and figures, please use this link (please enable cookies, or cut and paste URL):
Link Not Available

MBoC offers the option to publish your work with immediate open access. Open access can increase the discoverability and

usability of your research. If you would like to publish your paper with immediate open access but did not select this option during initial submission, please contact us at mbc@ascb.org..

Authors of Articles and Brief Communications whose manuscripts have returned for minor revision ("revise only") are encouraged to create a short video abstract to accompany their article when it is published. These video abstracts, known as Science Sketches, are up to 2 minutes long and will be published on YouTube and then embedded in the article abstract. Science Sketch Editors on the MBoC Editorial Board will provide guidance as you prepare your video. Information about how to prepare and submit a video abstract is available at www.molbiolcell.org/science-sketches. Please contact mboc@ascb.org if you are interested in creating a Science Sketch.

Thank you for submitting your manuscript to MBoC. Please do not hesitate to contact this office if you have any questions.

Sincerely,

Eric Baker
Journal Production Manager
MBoC Editorial Office
mbc@ascb.org

Reviewer #1 (Remarks to the Author):

E24-02-0058
Increased Intracellular pH is Sufficient to Induce Autophagic Cell Death
Jobelle Peralta1,2, Blake DuPriest1, Daniel Orozco1, Juan Reyna Pacheco1, Laura Martins1, Rachel Ann Soriano1, Alan Wong1, Ramy Wong1, Bree Grillo-Hill1

General.
The current MS uses Drosophila genetics and eye morphology and cell biology to support their thesis that increased intracellular pH (increased pHi, i.e., alkalinization) is the causal driver of this through the autophagy pathway. This reviewer would tend to agree that the autophagy pathway is in play; however, the explicit link to alkalinization or even altered pHi is not demonstrated. Key experiments to demonstrate cause and effect are needed (noted below). Either this link should be made or the MS recast as a more cell biology oriented MS.

Major.
1) pHi increase is causative of autophagy.
This is the thesis of the MS based on the results of Grillo-Hill et al. eLife 2015;4:e03270. (DOI: 10.7554/eLife.03270). As noted below, I believe there are short-comings in that paper, but here I will focus on the causal link of alkalinization causes autophagy. Simply put, this MS does not test that thesis
a. as noted below, there are NO pHi measurements in the MS, making it impossible to establish a causal link
b. Authors have not attempted to manipulate pHi with non-specific targeting of the fly eye to determine if pHi increase itself elicits this response
c. Authors do not know what genetic manipulations in Figures 3 and 4 do to pHi. This becomes crucial to support their current thesis as Figure 3 implicated apoptosis (autophagy) based on the partial ocular phenotype of H99 in the background of GMR>DNhe2 vs. GMRGAL4 x UAS-H99. We do not know IF H99 manipulation changes pHi or just the morphologic phenotype. While I agree it is likely, it is not demonstrated experimentally.
d. Discussion of H99 effected (downstream genes: grim, rpr and hid) would seem to be needed. Also note that only hid is even detectable in FlyAtlas [~2 FPKM in eye]. It is unclear what this might mean for the overall pathway stressed by the authors.
e. Similarly, with UAS-p35 crosses, we do not know that the lack of a morphologic phenotype is due to no or insignificant pHi changes.
f. Fig 4 - more documentation and pHi measurements are needed. According to FlyAtlas (1 or2), the levels of Atg1 (modest, 15 FPKM), Atg7 (low, 4-5 FPKM) and Atg8a (high, ~210) are not remotely similar in the larval brain or adult eye. In this context, it seems unclear why the modest expression of Atg1(3) has the most obvious morphologic phenotype. At minimum, the authors need to explain this.

2) pHi assessment
a. (p5, beginning of Results/Discussion) states: "To determine how increased pHi impacts tissue size in the Drosophila eye, quantitative analysis of the area of adult eyes was performed."
However, according to the Methods, pHi measurements were performed in this study. It should also be noted that pHi

measurements in the eLife 2015 paper, were not properly calibrated, i.e., most of the GMRGAL4>DNhe2 measurements and calculated pHi's are above the top end of their pH 7.5 range. This means that while pHi is almost certainly higher, we do not really know what the pHi range is. This becomes important because almost any animal cell type will overtly die if resting pHi is chronically >7.8.
b. Similarly Fig 2 title claims "...with higher pHi"
c. If the authors merely elevate pHi chemically (e.g., chronic NH4Cl or similar), what happens? This approach should distinguish between specific vs. generalized cell death

3) Cell Count assessment
a. How are the 3 different counts of cells in any given experiment handled for "N" total number of cells?
b. Are the 3 different "readers" always the same for each reported experiment?
c. Some sampling of reader1, reader2 and reader3 should be included as we do not know how these counts differ (could be as supplemental), i.e., what is "observer bias?"

4) Imaging details
a. Presumably the Zeiss LSM 700 confocal microscope is connected to a monochrome camera, but the type of camera should be specified as indicated in "high-resolution images of Drosophila eyes."
b. Unclear that normalizing to Hoechst 33342 channel is appropriate. Authors should explain why that this is done and provide some evidence that is biologically meaningful. Have the authors maintained individual channel settings for both photograph and images used for analysis? Even if the above normalization is shown to be appropriate, changing setting at all during imaging for the various channels could lead to artifacts. This is all "pre-FIJI" analysis that should be controlled.

5) Figure Notes
a. Fig 3 C should include all the groups in Fig 3A

Minor.
• Be consistent with Drosophila genetics and phenotypes. We do not need the detail found in Methods, but the non-fly audience needs to be able to understand the drivers (Gal4 line(s)) and targets (UAS lines). For example, GMRGAL4 would typically be GMR(italics)-Gal4 and UAS as UAS-H99, with the F1 cross GMR>H99, etc. Please standardize throughout

Reviewer #2 (Remarks to the Author):

see attached.

SJSU SAN JOSÉ STATE UNIVERSITY

Bree K. Grillo-Hill, Ph.D.
Associate Professor
Dept. of Biological Sciences

San José State University
One Washington Square
San José, CA 95192

TEL: 408-924-4890
bree.grillo-hill@sjsu.edu

The California State University:
Chancellor's Office
Bakersfield
Channel Islands
Chico
Dominguez Hills
East Bay
Fresno
Fullerton
Humboldt
Long Beach
Los Angeles
Maritime Academy
Monterey Bay
Northridge
Pomona
Sacramento
San Bernardino
San Diego
San Francisco
San José
San Luis Obispo
San Marcos
Sonoma
Stanislaus

April 19, 2024

Dr. Sergio Grinstein
Monitoring Editor
Molecular Biology of the Cell

Dear Dr. Grinstein,

We are submitting a revised manuscript now titled "*Drosophila Nhe2* Over-expression Induces Autophagic Cell Death". We thank the reviewers for their time reviewing this work, and their constructive comments and suggestions to improve our manuscript. Reviewer 2 praised it as "a new and significant finding" and said "the data and conclusions are convincing." Reviewer 1 "tends to agree that the autophagy pathway is in play". We are glad that both reviewers support resubmission with minor revisions.

We are submitting a revised manuscript that adds significant points of clarification and additional information based on reviewer comments and suggestions. Our revision includes editorial changes to address Reviewer 1's concerns about the conflation of increased intracellular pH with *DNhe2* over-expression. We added rationale to the introduction, and restricted discussion of increased pHi as the probable mechanism to the introduction and discussion sections. We also addressed Reviewer 2's comments regarding our figures, and revised and standardized our notations and labels to better communicate genotypes, statistical significance, and added additional detail to figure legends.

Our revision addresses all reviewer concerns, and below we include a point-by-point response to each reviewer concern. We believe that this is a stronger submission and hope that it is suitable for publication in *Molecular Biology of the Cell*.

Sincerely,

Bree K. Grillo-Hill, Ph.D.

<u>Point-by-point responses to reviewer comments:</u>

Reviewer #1:

General.

The current MS uses Drosophila genetics and eye morphology and cell biology to support their thesis that increased intracellular pH (increased pHi, i.e., alkalinization) is the causal driver of this through the autophagy pathway. This reviewer would tend to agree that the autophagy pathway is in play; however, the explicit link to alkalinization or even altered pHi is not demonstrated. Key experiments to demonstrate cause and effect are needed (noted below). Either this link should be made or the MS recast as a more cell biology oriented MS.

We thank this reviewer for their thorough review of our manuscript. Their comments helped us identify areas of the text that required clarification and improved explanation, and we appreciate their time and work in improving our manuscript. Overall, we appreciate their comment regarding the explicit link between autophagy and increased pHi. We have revised the introduction to emphasize our rationale, including discussing in detail data from our previous publications that suggests that increased pHi through *DNhe2* over-expression is responsible for the rough eye phenotype. We also edited the text to throughout the results section to clarify that over-expression of *DNhe2* (rather than increased pHi) is the experimental manipulation used. Finally, we restricted discussion of increased pHi as the probable mechanism (based on our data and previously published data) to both the introduction and discussion sections.

Major.

1) pHi increase is causative of autophagy.

This is the thesis of the MS based on the results of Grillo-Hill et al. eLife 2015;4:e03270. (DOI: 10.7554/eLife.03270). As noted below, I believe there are short-comings in that paper, but here I will focus on the causal link of alkalinization causes autophagy. Simply put, this MS does not test that thesis

> *a. as noted below, there are NO pHi measurements in the MS, making it impossible to establish a causal link*
>
> We thank this reviewer for their comment, and we have made revisions throughout the manuscript to address this concern. In our eLife 2015 paper, we showed that the retinal patterning errors that we observe with over-expression of *DNhe2* are in fact due to the ion transport function of the protein, rather than one of its other functions. Specifically, using the mammalian transport-dead E266I NHE1 mutation as a template, we identified the orthologous residue in *Drosophila* DNhe2 (E358I), and generated transgenic flies carrying this predicted "transport dead" mutant. We found that when we overexpressed the transport dead mutant, $DNhe2^{E358I}$, there was no change in pHi and no visible phenotypes in the *Drosophila* eye. We have revised the introduction (p. 3, 4) to emphasize this rationale, the prior results that show rough eye phenotype arises from ion transport activity of DNhe2, and to clarify that over-expression of *DNhe2* is the experimental manipulation we use throughout the results section (p. 6, 7). We restricted discussion of increased pHi as the probable mechanism to the introduction and discussion sections.
>
> *b. Authors have not attempted to manipulate pHi with non-specific targeting of the fly eye to determine if pHi increase itself elicits this response*
>
> Thank you for the suggestion. We agree that the proposed experiment would strengthen the causal connection between increased pHi and autophagy phenotypes. However, we lack alternative experimental approaches to robustly manipulate pHi *in vivo* in *Drosophila*. In addition to *DNhe2* overexpression (used in this manuscript), only one other tool has been shown to alter pHi *in vivo*, which is RNAi-mediated knock-down of *Drosophila* anion exchanger 2 in the *Drosophila* ovary (Benitez, et.al. 2019). There are not any published methods to non-specifically increase pHi in *Drosophila* over a sufficiently long time period to evaluate molecular markers of autophagy (often reported to take at least several hours; doi: 10.3390/toxics11080682 and DOI:https://doi.org/10.1016/j.devcel.2004.07.005). Our unpublished experiments with growing *Drosophila* on NH_4Cl-containing media or incubating *Drosophila* retinae or cell lines in NH_4Cl did not result in altered pHi.

c. Authors do not know what genetic manipulations in Figures 3 and 4 do to pHi. This becomes crucial to support their current thesis as Figure 3 implicated apoptosis (autophagy) based on the partial ocular phenotype of H99 in the background of GMR>DNhe2 vs. GMRGAL4 x UAS-H99. We do not know IF H99 manipulation changes pHi or just the morphologic phenotype. While I agree it is likely, it is not demonstrated experimentally.

We thank the reviewer for this comment. We did not perform pHi measurements in this work as we lack access to live-cell-imaging microscopy resources that enable these experiments. Our measurement in these experiments was cell number in pupal eyes, which is a well-established system for studying cell death. We clarified that over-expression of *DNhe2* is the experimental manipulation (p. 6), and restricted discussion of increased pHi as the probable mechanism to the introduction and discussion sections of the revised manuscript.

d. Discussion of H99 effected (downstream genes: grim, rpr and hid) would seem to be needed. Also note that only hid is even detectable in FlyAtlas [~2 FPKM in eye]. It is unclear what this might mean for the overall pathway stressed by the authors.

We thank the reviewer for raising this point, and we expanded our discussion of the *H99* deletion in the manuscript (p. 6). We used *H99* in our work because it has historically been used to completely block retinal apoptotic cell death induced by genes including *Dronc* (DOI 10.1074/jbc.M002935200), *DIAP1* (doi.org/10.1038/sj.cdd.4401538) and *argos* (doi.org/10.1038/sj.cdd.4400398). Together with our other data, we interpret this to mean that blocking apoptosis does not fully suppress the morphological defects caused by over-expression of *DNhe2*.

e. Similarly, with UAS-p35 crosses, we do not know that the lack of a morphologic phenotype is due to no or insignificant pHi changes.

We thank the reviewer for raising this point, and again answer that our measurement in these experiments was cell number, and not pHi. We believe that p35 over-expression is not likely to alter pHi, as it has a well characterized mechanism of covalent binding directly to caspases within the active site, thus preventing target protein cleavage (DOI: 10.1038/35068604). There are no published reports of altered pHi with caspase inhibition.

f. Fig 4 - more documentation and pHi measurements are needed. According to FlyAtlas (1 or2), the levels of Atg1 (modest, 15 FPKM), Atg7 (low, 4-5 FPKM) and Atg8a (high, ~210) are not remotely similar in the larval brain or adult eye. In this context, it seems unclear why the modest expression of Atg1(3) has the most obvious morphologic phenotype. At minimum, the authors need to explain this.

We thank the reviewer for pointing out the FlyAtlas expression data. It is important to note that the limited time points and tissue types contained in the FlyAtlas (retinal tissue only at adult stages) do not overlap with our data, which show missing cells at larval and pupal stages of retinal development. It is also worth noting that transcript levels often correlate poorly with protein levels (doi: 10.1039/b908315d), and for many proteins, abundance and activity are not directly correlated due to post-translational modification, allosteric regulation and environmental factors including pHi. We chose to quantify cell number in our $Atg1^3$ heterozygotes as these effects were the most robust and reproducible. We cannot rule out that this may be due to variable knock-down in the *Atg7* or *Atg8a* RNAi lines.

2) pHi assessment

a. (p5, beginning of Results/Discussion) states: "To determine how increased pHi impacts tissue size in the Drosophila eye, quantitative analysis of the area of adult eyes was performed."
However, according to the Methods, pHi measurements were performed in this study. It should also be noted that pHi measurements in the eLife 2015 paper, were not properly calibrated, i.e., most of the GMRGAL4>DNhe2 measurements and calculated pHi's are above the top end of their pH 7.5 range. This means that while pHi is almost certainly higher, we do not really know what the pHi range is. This becomes important because almost any animal cell type will overtly die if resting pHi is chronically >7.8.

In reference to the comment “pHi measurements were performed in this study”, we do not in fact perform pHi measurements in the current study. We have revised the manuscript to clarify that over-expression of *DNhe2* is the experimental manipulation, and restricted discussion of increased pHi as the proposed molecular driver to the introduction and discussion sections.

The critique in the rest of this section is outside the scope of the current paper, as it critiques previously peer-reviewed and published work that is not included in the current manuscript. Furthermore, we are in agreement with the reviewer’s overall conclusion that the 2015 eLIFE paper showed that over-expression of *DNhe2* increases intracellular pH in the fly eye.

Finally, we wish to reinforce a point the reviewer makes in the final point in this section that *“almost any animal cell type will overtly die if resting pHi is chronically >7.8”*. We agree that this is a long-standing observation with incompletely characterized molecular mechanism for the mode of death. Critically, in our current study, our results suggest that cell death with prolonged increased pHi acts specifically through the autophagy pathway.

b. Similarly Fig 2 title claims "...with higher pHi"

We have revised the manuscript to clarify that over-expression of *DNhe2* is the experimental manipulation, and restricted discussion of increased pHi to the introduction and discussion sections.

c. If the authors merely elevate pHi chemically (e.g., chronic NH4Cl or similar), what happens? This approach should distinguish between specific vs. generalized cell death

As stated above in response to critique 1b, only two manipulations have been shown to increased pHi in *Drosophila in vivo*: *DNhe2* overexpression (used in this manuscript), and RNAi-mediated knock-down of *Drosophila* anion exchanger 2 in the *Drosophila* ovary (Benitez, et.al. 2019). There are not any published methods to chemically increase pHi in *Drosophila* retinal tissue, and our attempts to develop such methods have so far been unsuccessful (unpublished data).

To the second point, the reviewer also makes a distinction between “*specific vs. generalized cell death”.* Unfortunately, without defining what those differences are expected to be, it was impossible to determine what the reviewer means by “specific” and “generalized”. Importantly, we used morphological analysis, genetic interaction studies, and genetically-encoded autophagy markers to determine that the observed cell death required proteins in the autophagy pathway. Taken together, our work suggests a cellular mechanism by which overexpression of *DNhe2* in the *Drosophila* eye leads to a cell death phenotype mediated by autophagy pathway members. By definition, this is a specific and molecularly defined cell death pathway (autophagy-mediated).

3) Cell Count assessment

a. How are the 3 different counts of cells in any given experiment handled for "N" total number of cells?

We thank the reviewer for this question, and have updated our methods section to reflect our practices (p. 10). After image acquisition and processing, at least three individuals perform counts, which are reconciled through discussion into a consensus cell count. All students were trained based on the following protocol: “Cell counts are performed by identifying a central ommatidium and its six nearest neighbors, and drawing a hexagon through the center of the cone cells in the six neighbors. Each cell completely contained in this hexagon counts as one cell, and cells that are partially contained count as one-half cell.” Individuals new to cell counting were given previously-analyzed datasets to confirm data concordance with experienced counters. Cell counts were performed by at least 3 different people for reproducibility, and any discrepancies were discussed to consensus. One individual eye imaginal disc or pupal eye are taken from each animal.

b. Are the 3 different "readers" always the same for each reported experiment?

We thank the reviewer for this query. We are a team of scientists at a PUI where the average lab tenure is 1-2 years. The data collection for this manuscript took more than 6 years, so the 3 readers were not always the same across data sets. The PI/corresponding author trains students, spot-checks data for new counters and participates in all data collection and analysis, which provides strong consistency across analyzed datasets.

c. Some sampling of reader1, reader2 and reader3 should be included as we do not know how these counts differ (could be as supplemental), i.e., what is "observer bias?"

We thank the reviewer for pointing out this, and have expanded our methods to better explain how we determine counts (p. 10). In short, there is no discrepancy in counts to report as the rare cell count differences are discussed to consensus with input and guidance from the PI/corresponding author (see 3a and b responses above).

4) Imaging details

a. Presumably the Zeiss LSM 700 confocal microscope is connected to a monochrome camera, but the type of camera should be specified as indicated in "high-resolution images of Drosophila eyes."

We appreciate the reviewer's attention to detail regarding our microscopy methodology. The Zeiss LSM 700 confocal microscope is a ready-built (not modular) system, and the only information provided about the camera is "High-resolution AxioCam microscope camera". We have updated the methods section to include this information.

b. Unclear that normalizing to Hoechst 33342 channel is appropriate. Authors should explain why that this is done and provide some evidence that is biologically meaningful. Have the authors maintained individual channel settings for both photograph and images used for analysis? Even if the above normalization is shown to be appropriate, changing setting at all during imaging for the various channels could lead to artifacts. This is all "pre-FIJI" analysis that should be controlled.

We appreciate this reviewer's thoughtful comments regarding our image acquisition parameters. We have added detail to the methods section to outline our approach (p.10), and provide context and justification for our normalization to Hoechst. We have also edited the methods to explicitly described that control and experimental images were acquired on the same day using identical acquisition settings (p. 10), which avoids the artifacts described by the reviewer.

5) Figure Notes

a. Fig 3 C should include all the groups in Fig 3A

We appreciate this suggestion, and have added additional text to the results to explain our rationale for only performing counts for the *p35 – DNhe2* genetic interaction. After we ruled out caspase involvement in the observed cell death, we chose to focus on identifying the mode of cell death as described in revised Figure 4.

Minor.

• Be consistent with Drosophila genetics and phenotypes. We do not need the detail found in Methods, but the non-fly audience needs to be able to understand the drivers (Gal4 line(s)) and targets (UAS lines). For example, GMRGAL4 would typically be GMR(italics)-Gal4 and UAS as UAS-H99, with the F1 cross GMR>H99, etc. Please standardize throughout

We appreciate the reviewer's comments and attention to detail. We have standardized our nomenclature throughout the revised manuscript.

Reviewer #2:

SUMMARY
The study by Peralta and colleagues investigates the underlying mechanisms of how changes in intracellular pH (pHi) can impact cell signaling, growth and differentiation of tissues, using development of the Drosophila eye as a genetically tractable model system. Patterning of the Drosophila eye (composed of 750 identical multicellular units called ommatidia) involves sequential phases of cell proliferation (larval), differentiation and regulated cell death (pupal) to achieve the adult eye configuration. Previous studies from this group (Grillo-Hill, B. K., et al. (2015) Elife) and others (Simons, M., et al. (2009). Nat. Cell Biol.) have identified the plasma membrane Na+/H+exchanger Dnhe2 as a critical component of this process, as dysregulation of Dnhe2 disrupts Wnt signaling, planar cell polarity and tissue architecture of the Drosophila eye.

Notably, earlier results showed that overexpression of Dnhe2 caused alkalinization of cells within the eye imaginal disc and induced tissue patterning errors in the ommatidia that manifested visibly as a rough eye phenotype in adult flies. This was associated with increased cell proliferation in the posterior region of the developing eye imaginal disc, yet unexpectedly resulted in decreased overall size of adult eyes. However, the underlying mechanisms were unresolved. In the present study, the authors found that while cone cells were unaffected, there were significant decreases in secondary and tertiary pigmented cells as well as bristle complexes of the ommatidia. Additional evidence (genetic manipulation of anti-apoptotic genes) excluded apoptosis as a contributing factor, and instead identified a previously unrecognized role for genes involved in autophagic cell death. Overall, this is a new and significant finding. The data and conclusions are convincing, though there are several minor concerns in the manuscript that need to be addressed as outlined below.

COMMENTS
The manuscript is generally well written and the interpretations are consistent with the data, although the concluding statement that their findings support "inhibiting autophagy as a therapeutic approach to cancer" is a bit of a stretch since cancerous cells were not examined in this study. Also, the manuscript was not carefully proof-read and contains several errors and omissions that need correcting.

1. There are no page numbers.

We thank this reviewer for pointing out this oversight, and added page numbers to our manuscript.

2. Page 3 (Introduction), it is stated that Drosophila Dnhe2 is the ortholog of the ubiquitous plasma membrane NHE1 isoform in mammals. However, this assertion conflicts with a previous study (Simons, M., et al. (2009). Nat. Cell Biol.) which claims—based on sequence alignments—that DNhe2 is the ortholog of the mammalian epithelial apical membrane NHE3 isoform. Please clarify.

We thank the reviewer for this question. We addressed this discrepancy with the 2009 Nat. Cell Biol paper in Figure 1A of our previously published 2015 eLIFE paper. Mammalian NHE1 has conserved domains that are only present in *Drosophila* Nhe2, including conserved sequences in the transmembrane domains, a lysine/arginine-rich region (KR motif) that facilitates membrane localization through phosphatidylinositol 4,5-bisphosphate (Putney and Barber, 2003), a conserved binding motif for the calcineurin homologous protein CHP (Lin and Barber, 1996; Pang et al., 2001), a functionally conserved glutamic acid residue in the transmembrane domain that is essential for H+ efflux (E358 in DNhe2 and E266 in human NHE1) and consensus sites for phosphorylation by Akt and ATM/ATR kinases in the C-terminus of both proteins.

3. In Fig. 1A, the genotype of each panel should be labeled. The control fly contains the white gene (W) mutation W1118 (left panel) that generates white-eyes, whereas the eyes in the middle and right panels are shades of red. Are the genotypes of the GMRGAL4+ and GMR>DNhe2 flies derived from W1118 in order to make a valid comparison of eye size? More description of the different genotypes is warranted for those not familiar with fly genetics. Fig. 1B, I don't understand the statistical depictions in the inset box. A single asterisk is indicated for W1118 and GMRGAL4/+ ($p < 0.05$), but different from what? What is the significance level for the double asterisk. What is the error representative of, SEM or SD? Include the ranges in the figure legend.

We thank the reviewer for this comment, and have added clarifications in the results section with additional rationale for the controls used (p. 5), and have added more detail in the revised figure legends. We also revised our figures for more consistent and clear communication of statistical significance, and added this detail to revised figure legends.

4. The images in Fig. 1C and 1D were stained with an antibody against Armadillo. It would help orient the reader if the nature of the Armadillo protein was described in the text (i.e. vertebrate homolog of -catenin).

We thank the reviewer for this comment, and have added the requested detail to our revised manuscript (p. 5).

5. Page 6. (Quinn et al. 2003). Not listed in reference section.

We thank the reviewer for catching this omission, and have added this reference in the revised reference section.

6. Fig. 3C, label is GMR>p35+DNhe2, differs from text GMR>DNhe2+p35.

We thank the reviewer for catching this error, and have standardized this. We standardized our nomenclature and labeling indicators across the revised text and figures.

7. Page 7. Last sentence of first paragraph refers to Figure 4D, but should be Figure 4E. Please place the asterisk symbols of Figure 4E (currently positioned at the outer upper extremity of the figure) closer to the respective data.

We thank the reviewer for this comment, and have made the requested revision. We standardized our statistical indicators across figures.

8. Figure 4G, statistical significance is missing.

We thank the reviewer for this comment, and have added the requested detail to our revised figure.

9. Page 7 (bottom); the discussion on autophagy and its controversial involvement in cell death is lacking citations.

We thank the reviewer for this request, and we have added references in our revised manuscript

RE: Manuscript #E24-02-0058R
TITLE: "Drosophila Nhe2 Over-expression Induces Autophagic Cell Death"

Dear Dr. Grillo-Hill:

Thank you for submitting a revised version of your manuscript addressing the reviewers' comments. I noted particularly your willingness to modify the conclusions to state that overexpression of Nhe2, rather than the resulting or expected change in pH, is responsible for the phenotypic outcomes observed.
I feel that the revised manuscript is now acceptable for publication in Molecular Biology of the Cell in its present form.

Sincerely,
Sergio Grinstein
Monitoring Editor
Molecular Biology of the Cell

Dear Dr. Grillo-Hill:

Congratulations on the acceptance of your manuscript! Thank you for choosing to publish your work in Molecular Biology of the Cell (MBoC).

Within 10 days, an unedited PDF of your manuscript will be published on MBoC in Press, an early release version of the journal. The date your manuscript appears on this site is the official publication date.

Your copyedited and typeset manuscript will be scheduled for publication in the next available issue of MBoC. Our production team will notify you when the page proofs of your paper are ready for your review.

MBoC offers the option to publish your paper with immediate open access. Open access can increase the discoverability and usability of your research. If you would like to publish your paper with immediate open access but did not select this option during initial submission, please contact the MBoC Editorial Office (mbc@ascb.org).

We invite you to submit images related to your accepted manuscript to be considered for the journal cover.
Cover images must contain color, TIF Format
Size image at 7.4" wide x 5.85" deep
RGB and CMYK color (please submit two separate files, one in each format)
300 dpi resolution

Please also send a 1-paragraph description of the image that a general audience could understand. Include with the caption the name and institution of the individual(s) the image should be attributed to. Please provide this description in a Word file. You can send the file via email or by your favorite file transfer service to mboc@ascb.org.

Authors of Articles and Brief Communications are also encouraged to create a short video abstract to accompany their article when it is published. These video abstracts, known as Science Sketches, are up to 2 minutes long and will be published on YouTube and then embedded in the article abstract. Science Sketch Editors on the MBoC Editorial Board will guide you as you prepare your video. Information about how to prepare and submit a video abstract is available at www.molbiolcell.org/science-sketches. Please contact mboc@ascb.org if you are interested in creating a Science Sketch.

We look forward to publishing your paper in MBoC.
Sign up for MBoC's new content alerts here https://tinyurl.com/3bcsyk3e

Sincerely,

Eric Baker
Journal Production Manager
MBoC Editorial Office
mbc@ascb.org
